# A Comparison by Milk Feeding Method of the Nutrient Intake of a Cohort of Australian Toddlers

**DOI:** 10.3390/nu8080501

**Published:** 2016-08-16

**Authors:** Jane Scott, Kristina Davey, Ellen Ahwong, Gemma Devenish, Diep Ha, Loc Do

**Affiliations:** 1 Collaboration for Evidence, Research and Impact in Public Health (CERIPH), School of Public Health, Curtin University, Perth 6102, Australia; 2 School of Public Health, Curtin University, Perth 6102, Australia; kristina.davey@postgrad.curtin.edu.au (K.D.); ellen.pearce@postgrad.curtin.edu.au (E.A.); gemma.Devenish@curtin.edu.au (G.D.); 3 Australian Research Centre for Population Oral Health, University of Adelaide, Adelaide 5000, Australia; diep.ha@adelaide.edu.au (D.H.); loc.do@adelaide.edu.au (L.D.)

**Keywords:** nutritional adequacy, diet, toddler, breastmilk, formula, iron

## Abstract

Breastfeeding is recommended beyond 12 months of age, but little is known about the contribution of breastmilk and infant formula to the nutritional intake of toddlers as they transition to a family diet in the second year of life. This study is a cross-sectional analysis of data collected from a birth cohort study in Adelaide, Australia. Dietary intake data were collected when children were approximately 1 year of age by an interviewer-administered multi-pass 24 h recall and a mother-completed 2 days food diary. Children were categorized according to their milk feeding method, i.e., breastmilk, infant formula, combination or other, and their nutrient intakes compared with recommended nutrient reference values. Complete data were available for 832 children, of which 714 had plausible energy intakes. Breastmilk and formula made a substantial contribution to the nutrient intake of those toddlers, contributing 28% and 34% of total energy, and 16% and 26% of protein intake, respectively when not drunk in combination. In general, Australian toddlers transitioning to the family diet consumed nutritionally adequate diets, although almost one quarter of all children and half of breastfed children with plausible intakes had iron intakes below the estimated average requirement, placing them at risk of iron deficiency.

## 1. Introduction

Breastfeeding is recommended beyond 12 months of age [1,2,3], but relatively little is known about the contribution of breastmilk and infant formula to the nutritional intake of toddlers as they transition to a family diet in the second year of life. Similarly, little is known about the nutritional content and adequacy of the diets of Australian children under the age of 2 years. While the Australian National Infant Feeding Survey studied this age group, its focus was on breastfeeding and age of introduction of complementary foods and it did not investigate what and how much children in this age group ate [4]. On the other hand, the 2011–2012 Australian National Nutrition and Physical Activity Survey (NNPAS) [5] and the 2007 Australian National Children’s Nutritional and Physical Activity Survey (NCNPAS) [6] only studied the diets of children aged 2 years and older. What data are available on the diets of Australian infants and toddlers aged less than 2 years come from a number of single-center or state-based studies of infants and toddlers of varying ages [7,8,9,10,11,12]. Relatively little is known about the diets of children as they enter their second year of life [9] and much of what we know about the diets of this age group comes from international studies [13,14,15].

The objectives of this paper were therefore to (1) estimate the usual intake of a cohort of Australian toddlers and to evaluate their diets relative to the Nutrient Reference Values (NRVs) for Australia and New Zealand [16]; (2) identify the contribution that breastmilk and formula make to the nutrient intake of toddlers; and (3) investigate the relationship between milk feeding method and nutrient adequacy.

## 2. Materials and Methods

### 2.1. Design

This study is a cross-sectional analysis of dietary data collected as part of the Study of Mothers’ and Infants’ Life Events Affecting Health (SMILE), a population-based longitudinal birth cohort study [17]. This study has recruited and is following a cohort of socioeconomically-diverse South Australian newborns from birth and into their third year of life. The primary health outcomes of this study are two related conditions: dental caries and obesity/overweight of young children.

### 2.2. Setting and Recruitment

In total, 2147 mothers and 2181 newborns, including 34 pairs of twins, were recruited from the three major maternity hospitals in Adelaide, Australia from July 2013 until August 2014. All new mothers who were sufficiently competent in English to be able to understand the description and instructions of the study were invited to participate. Those mothers who indicated their intention to move out of the greater Adelaide area within a year were excluded.

Mothers were recruited, usually within 48 h of giving birth, from the postnatal wards of the participating hospitals. Those agreeing to participate in the study were invited to complete a baseline questionnaire designed to collect mother and family-related information including socio-demographic details such as age, income, education, occupation and postcode. Further details of sample size and recruitment procedures have been reported elsewhere [17].

### 2.3. Ethical Considerations

The study was approved by the Southern Adelaide Clinical Human Research Ethics Committee (HREC/50.13, approval date: 28 February 2013) and the South Australian Women and Children Health Network (HREC/13/WCHN/69, approval date: 7 August 2013), and received clinical governance clearance from the three participating maternity hospitals. Signed informed consent was obtained from mothers who were advised that their participation was voluntary and that they could withdraw at any time without prejudice.

### 2.4. Collection and Handling of Dietary Data

Once their child had reached 12 months of age, mothers of the 1919 infants remaining in the study were mailed a food diary and a cover letter explaining that a member of the research team would telephone them to collect a 24 h dietary recall (24 HDR) of their child’s intake and explain how to complete the food diary. The 24 HDR was conducted via a telephone interview by a trained dietitian using the five-step multi-pass method [18]. At the end of the 24 HDR interview mothers were allocated two days in the following week to record their child’s food intake in the food diary. The days allocated ensured that, together with the 24 HDR, three nonconsecutive days (2 week days and one weekend day) of dietary intake over a 10-day period were recorded. The food diary booklet contained instructions for recording their child’s intake and included a detailed example of a one-day food record, as well as photos of food portion sizes and examples of household measures (cups, bowls and spoons), to help mothers estimate amounts consumed when completing both the 24 HDR and the food diary.

The dietary data were entered into FoodWorks^®^ version 8 (Xyris Software) and analyzed using the AUSNUT 2011–2013 food composition database [19]. Foods consumed by children but not included in the AUSNUT database were added utilizing nutrient information gathered from the nutrition information panels of product labels and manufacturer websites. The 24 HDR and the two-day food diary were entered together as a food record by a team of four nutritionists. For quality management purposes, standardization training was conducted and data were entered following a detailed data entry protocol. Cross-checking between the 24 HDR and food diary was conducted for data clarification purposes—for example, where a type of bread (e.g., wholegrain) had been collected as part of the 24 HDR but not recorded by the mother in the food diary.

Breastmilk intake was estimated using the method employed for this age group in the UK 2011 Diet and Nutrition Survey of Infants and Young Children (DNSIYC) [14]. Breastfeeds were recorded in minutes and the amount of milk consumed was calculated as 10 g/min to a maximum of 100 g per feed, as the contribution to nutrient intake after 10 min of breastfeeding is considered minimal in this age group [20]. If within 30 min of the start of the previous feed a second breastfeed was started, it was not considered a new feed, and the breastfeeding time was added to the previous feed to a maximum of 10 min [14]. If the child was breastfed for less than two minutes, this was not considered long enough to contribute to their nutrient intake and was not included [14].

Children were grouped initially into four categories of milk feeding method on the basis of whether or not they had consumed breastmilk and/or formula on one or more days. Formulas included infant formula, defined as suitable for children up to 12 months, and toddler formula. The ‘breastmilk group’ included toddlers who had received breastmilk but no formula. The ‘formula group’ included toddlers who had received infant or toddler formula but no breastmilk. The ‘combination group’ included toddlers who had received both breastmilk and infant or toddler formula. Children in these first three groups may also have consumed (usually in small amounts) animal milk or animal milk substitutes in addition to breastmilk and/or formula. Children in the ‘other’ group had consumed neither breastmilk nor infant or toddler formula.

### 2.5. Statistical Analysis

Once entered into FoodWorks^®^ version 8 (Xyris Software, High Gate Hill, Qld, Australia), data were downloaded into an Access database (Microsoft Office, 2013) and imported into SPSS version 22 (IBM SPSS Statistics for Windows, Armonk, NY, USA) for statistical analysis. Descriptive statistics were run to identify outliers and improbable intakes of weight of food, energy and macronutrients, and data were checked and cleaned appropriately. As the child’s current weight was unknown, a plausible energy intake was estimated for each child using a sex specific estimated energy requirement (EER) for a reference child of the participant’s age [16]. The child’s date of birth and the date when the 24 HDR was completed was used to calculate their age. The degree of under and over-reporting was assessed by calculating the ratio of reported energy intake to the EER for each child. Children were deemed to have an implausible intake if they had an average daily energy intake below 0.54 or above 1.46 for their age and sex specific reference EER [7].

Descriptive statistics, including mean values and standard deviations as well as 25th and 75th percentiles, were derived for children with plausible intakes for nutrient intakes averaged across the three days. The data were then transformed to determine the proportion of children with inadequate and excessive intakes compared with the NRVs for children aged 1–3 years [16]. Inadequate intakes were considered to be any value below the Estimated Average Requirement (EAR) and excessive intakes were considered to be any value above the Upper Limit of intake (UL), where relevant.

The mean intake of energy, macronutrients and those micronutrients for which more than 10% of the total sample had inadequate or excessive intakes were reported for each milk feeding group. A one-way ANOVA with a *post hoc* Bonferonni test was used to determine any significant between-group differences in mean intake of each nutrient. The percentage contribution of breastmilk and formula to total intake of energy and nutrients was determined for each group. Due to the small numbers of children with inadequate intakes in some groups, those children who received any formula whether in combination with breastmilk or not were collapsed into a single group for further analysis. The proportion of children with inadequate or excessive intakes in each of these three milk feeding group was then determined. Multivariate binary logistic regression, adjusting for maternal age, education and infant sex, was employed to determine the relationship of milk feeding group and inadequate or excessive intakes of nutrients. A *p*-value of < 0.05 was considered to be statistically significant for all analyses.

## 3. Results

**Figure A1:**
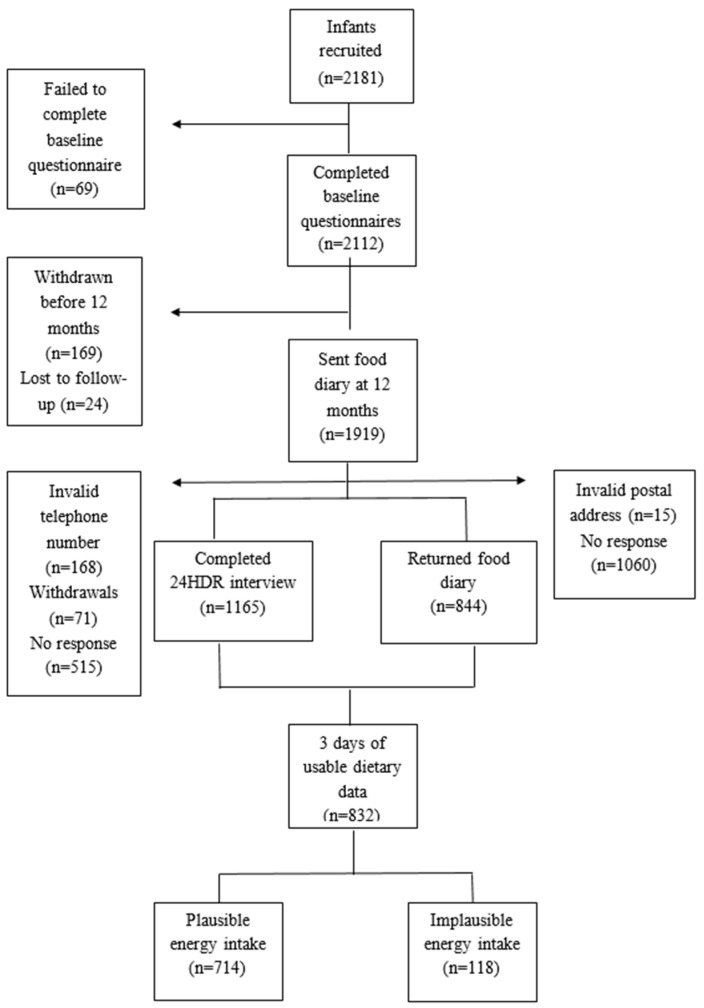
Participant Flow Chart.

Of the 1919 children who were mailed a food diary, 1165 had a completed 24 HDR interview (61% response rate) and 844 had returned food diaries (44% response rate). Usable, complete 3 day records were available for 832 children (43%). The participant flow chart is presented in Figure A1.

### 3.1. Participant Characteristics

Mothers who provided baseline data but did not provide complete dietary data at 12 months were younger (*p* < 0.001), less educated (*p* < 0.001), more socially disadvantaged (*p* < 0.001) and more likely to have been born outside of Australia (*p* < 0.001). However, due to the deliberate over-recruitment of disadvantaged women into the SMILE study [17], women who provided complete dietary data were generally representative of the socioeconomic profile reported by the Pregnancy Outcome Unit for South Australian for births in 2013 with regards to parity and country of birth, but our sample consisted of fewer younger (<25 years) and overweight or obese mothers [21] (Table S1).

The mean age of participant children was 13.1 ± 0.8 months and 54.7% were boys. In total, 118 children had implausible energy intakes (3 under-reporters and 115 over-reporters) and those 714 children with plausible energy intakes represent the analysis population in this study. The characteristics of mother-child dyads with and without plausible dietary data are presented in Table 1. There was no significant association between over-reporting and any of the maternal or child characteristics investigated, with the exception of milk feeding method.

### 3.2. Nutrient Intake of Children

#### 3.2.1. Comparison of Nutrient Intakes with Nutrient Reference Values

**Figure 1:**
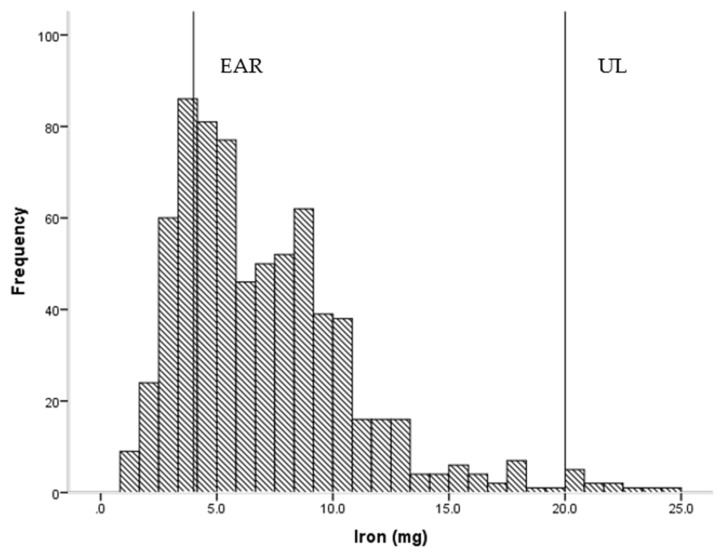
Frequency of intake of iron (*n* = 714) showing estimated average requirement (EAR = 4 mg) and upper limit of intake (UL = 20 mg).

**Figure 2:**
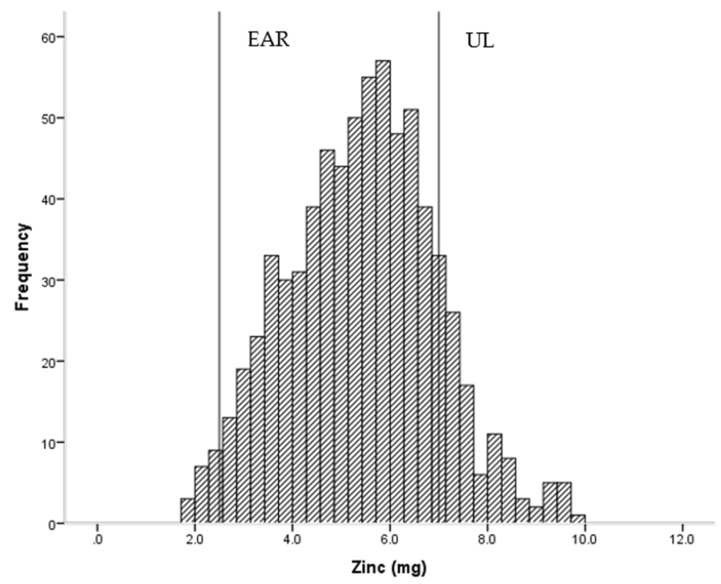
Frequency of intake of zinc (*n* = 714) showing estimated average requirement (EAR = 2.5 mg) and upper limit of intake (UL = 7 mg).

Overall, children had adequate daily intakes of energy, protein and micronutrients, with a few notable exceptions (Table 2). Roughly one in ten children had intakes below the EAR for vitamin C (14.0%), thiamin (14.4%) and calcium (12.6%), while just over two out of ten (22.5%) had intakes below the EAR for iron (Figure 1). Conversely, 18.2% and 14.1% had intakes which exceeded the UL for sodium and zinc (Figure 2), respectively. Dietary fibre intake was generally low in this group.

#### 3.2.2. Comparison of Nutrient Intakes by Milk Feeding Method

The majority of all 832 children were still receiving breastmilk (*n* = 334, 36%) and/or formula (*n* = 374, 53%); only 26 children (4%) were consuming toddler formula. Of those with plausible energy intakes, 260 children (36%) were consuming breastmilk and 334 children (47%) were consuming formula. Children were categorized according to whether or not they consumed breastmilk and/or formula and the mean intakes of energy, macronutrients and those micronutrient for which more than 10% of children had inadequate or excessive intakes were compared (Table 3). There were a number of significant between-group differences for all nutrients but in general children who did not drink either breastmilk and/or formula had significantly higher intakes of energy, protein, sodium, calcium and a lower intake of vitamin C than the other groups. There was no significant difference in the mean protein and energy intakes of those children in the breastmilk group compared with those in the formula group, although formula made a greater contribution to the total protein (26% vs. 16%) and energy intake (34% vs. 28%) of children than did breastmilk. Those children who drank only breastmilk had significantly lower intakes of zinc than all other groups, while those children who drank only formula had significantly higher intakes of calcium than those children who consumed breastmilk either with or without formula, and significantly higher intakes of zinc and iron than all other groups. Formula contributed 31% and 50% of the total iron intake in those who drank formula with or without breastmilk, respectively.

### 3.3. Relationship of Milk Feeding Method and Adequacy of Selected Nutrients

Due to the small number of children with inadequate intakes in some groups, those children who received any formula, whether in combination with breastmilk or not, were collapsed into a single group. Those children who were in the breastmilk-only group were significantly more likely than the other milk feeding groups to have intakes of calcium, iron, and thiamin below the EAR (Table 4). Children who drank neither breastmilk nor formula were almost 10 times more likely (AOR 9.64, 95% CI 5.27–17.62) than the breastmilk-only group to have intakes of vitamin C below the EAR, but children who drank formula were less likely (AOR 0.23, 95% CI 0.09–0.59) to have inadequate vitamin C intakes. Almost half (48.8%) of the children in the breastmilk-only group had iron intakes below the EAR while children who consumed formula were less likely (AOR 0.04, 95% CI 0.02–0.07) to have inadequate iron intakes than those who drank breastmilk. Conversely, children in the breastmilk-only group were less likely than all other groups to have intakes of zinc above the UL and less likely than the group that drank neither breastmilk nor formula to have sodium intakes above the UL.

## 4. Discussion

In this study we have described the diets of a cohort of Australian toddlers aged approximately 1 year, an age by which they should have transitioned predominantly to family foods. More than one half and one third of children continued to consume formula or breastmilk, respectively. These ‘milks’ contributed up to roughly one third of their total energy intake and one quarter of their protein intake when consumed either alone or in combination. A relatively high prevalence of formula feeding (32%) has been reported in another Australian cohort of slightly older toddlers (12–16 months) [9] and 38% of children aged 12–18 months in the UK-based DNSIYC were receiving formula of some kind [14]. The higher prevalence of formula use in our study can be attributed to the younger age of this birth cohort, which had a mean age of 13.1 months at the time that dietary intake was assessed. The Australian Infant Feeding Guidelines (AIFG) encourage the continuation of breastfeeding beyond the first year of life, and while it is recommended that cow’s milk not be given as a main drink to infants under the age of 12 months, this restriction is lifted once children enter their second year of life. Furthermore, the AIFG specifically state that “special complementary foods or milks for toddlers are not required for healthy children” [1] (p. 89).

It is unclear why contemporary Australian mothers continue to feed their children formula beyond 12 months of age. However, the advertising of toddler formulas has become increasingly prevalent [22] since Australia became a signatory to the International Code of Marketing of Breastmilk Substitutes (WHA 34.22 1981) which prohibits the advertising of infant formulas. With both products sharing common visual packaging elements such as colour, shape, typeface and logo, toddler milk advertisements appear to function as *de facto* infant formula advertisements with most women not being able distinguish between toddler and infant formulas and referring to both as ‘formula’ [23,24]. While most toddlers in this study were still consuming ‘infant’ versions of formula the advertising of toddler formula may have promulgated the perception amongst mothers that formulas of any kind are beneficial to the health of toddlers and are essential to meet the needs of developing toddlers that cannot be met by cow’s milk and family foods.

In general, toddlers in this study consumed diets that either met or exceeded their nutritional requirements, with relatively small numbers failing to meet the EAR for any nutrient. A notable exception was our finding that roughly one in five toddlers consumed diets below the EAR of 4 mg/day for iron [16] which placed them at risk of iron deficiency. This is consistent with the findings of a recent Australian study of slightly older toddlers (mean age: 19.6 months) which reported a similar mean iron intake of 6.6 mg/day and that 18.6% of toddlers had inadequate iron intakes [8]. Comparison with international studies is difficult due to differences in the nutrient recommendations between countries and methodological differences in the way in which dietary data are collected. Nevertheless, just over one in 10 (13%) of UK children aged 12–18 months had iron intakes below the Lower Recommended Nutrient Intake (LRNI) of 3.7 mg/day [25]. When this slightly lower cut point was applied to our data, 17.8% of the SMILE cohort had intakes below the UK LRNI for iron. In comparison, less than 1% of toddlers aged 12 to 23 months in the US Feeding Infants and Toddlers Survey (FITS) reportedly consumed diets below the EAR for iron, which for the USA is set lower, at 3 mg/day for this age group [13]. Nevertheless, when the USA cut-off point was used, 8.3% of the SMILE cohort had an average daily iron intake below this level, which remains appreciably higher than that reported in the FITS.

Children in this study who consumed formula, either with or without breastmilk, were unlikely to have inadequate iron intakes. Conn et al. reported a significant difference in the average daily iron intake of breastfed and non-breastfed infants in a younger cohort of children aged 9 months (6.3 mg/day vs. 11.9 mg/day, *p* < 0.001) [7]. Infant formulas are required by law to be fortified with iron, and in our study toddlers consuming formula either in combination with breastmilk or alone received one third to one half of their iron from formula, respectively. On the other hand, while the iron in breastmilk is highly absorbable, the overall levels are low [26] and breastmilk contributed only 3% of the total iron intake in those who were breastfed and not consuming formula. The Euro-Growth study investigated the determinants of iron status in 12-month-old infants and reported that the most important factor positively associated with iron status was the duration of feeding of iron-fortified formula [27].

Foods that children transition to are often poor sources of iron or they may consume low amounts of those foods which are rich in iron [9]. For instance, Byrne et al. reported that the median intake of meat and alternatives amongst a group of Australian toddlers aged 12–16 months was 56 g/day and one quarter had intakes of 28 g/day or less, which is well below the recommended daily intake for this age group of a single 65 g serve of this food group [28]. A low intake of this food group, which is the primary source of iron in the diets of toddlers, is likely to be more of an issue for breastfed toddlers than those who continue to consume appreciable amounts of either infant or toddler formula. Almost one half of children who were breastfed and not consuming formula and one third of children who were not consuming either formula or breastmilk had iron intakes below the EAR, compared with less than 5% of children consuming formula.

While roughly one in ten of all children had calcium and thiamin intakes below the EAR, almost one third of children who were breastfed without receiving formula failed to meet the EAR for both these nutrients. Children from the other milk feeding groups were significantly less likely than those in the breastfed only group to fail to meet the EAR for calcium and thiamin. As with iron, formula contributed a larger proportion of the overall intake of both calcium (50%) and thiamin (36%) in the formula only group than did breastmilk (28% and 11%, respectively) in the breastmilk-only group. This finding is partly explained by the fact that the formula group consumed on average more formula (443 g) than the breastmilk group consumed breastmilk (352 g), and that formula is typically fortified with vitamins and minerals at levels which exceed the highly variable levels of these nutrients found in breastmilk.

The primary limitations of this study are that usual intake of nutrients was estimated by summing and averaging the three days of intake, though this method does not adjust for the day-to-day variability in intake within individuals, which can be achieved with the use of specialist software that was not available to the researchers [29]. Nevertheless, this method is likely to provide more reliable estimates of usual nutrient intake than estimates derived from a single 24 h recall. While less than half of the mothers returned complete dietary data on the intake of their child, the deliberate oversampling of participants from disadvantaged groups means that the sample of women and children in this study are generally representative of the population from which they were drawn.

## 5. Conclusions

This is one of the first studies to report the contribution of breastmilk and formula to the diets of Australian children as they transition to family foods at the beginning of their second year of life. While the majority of children had intakes which met or exceeded their nutrient requirements, those children who consumed breastmilk only as their milk feed were at greater risk of having intakes below the EAR for iron, calcium and thiamin. This finding should, however, be interpreted with caution and women should continue to be encouraged to breastfeed their children beyond 12 months of age. However, it is possible that breastfeeding mothers may have a misplaced faith in the nutritional superiority of breastmilk over formula and cow’s milk as they transition to the family meal. All mothers, regardless of the milk they feed their child, must be educated and encouraged to feed their infant a varied and high-quality diet with adequate serves of the core food groups. In particular, health professionals should advise mothers of the importance of incorporating iron- and calcium-rich complementary foods into their child’s diets, both of which are important nutrients required for adequate growth and development of toddlers.

## Figures and Tables

**Table 1:** Characteristics of mother–child dyads with complete dietary data and of the subsets with plausible and implausible energy intake.

	Total		Plausible		Implausible		
	(*n* = 832)		(*n* = 714)		(*n* = 118)		
**Maternal characteristics**	*n*	%	*n*	%	*n*	%	*p* ^a^
Maternal age at birth (years)							
<25	74	8.9	62	8.7	12	10.3	0.583
25–29	259	31.2	227	31.9	32	27.4	
≥30	496	59.8	423	59.4	73	62.4	
Maternal education completed							
School/vocational	358	43.4	310	43.7	48	41.0	0.585
Some university and above	468	56.7	399	56.3	69	59.0	
IRSAD score ^b^							
Deciles 1–2	120	14.5	104	14.7	16	13.7	0.941
Deciles 3–4	175	21.2	147	20.7	28	23.9	
Deciles 5–6	172	20.8	147	20.7	25	21.4	
Deciles 7–8	161	19.5	140	19.7	21	17.9	
Deciles 9–10	198	24.0	171	24.1	27	23.1	
Mother’s country of birth							
Australia and N. Zealand	613	74.1	519	73.1	94	80.3	0.061
Asia—other	57	6.9	35	4.8	5	1.7	
India	50	6.0	47	6.6	3	2.6	
China	36	4.4	34	3.2	2	6.8	
UK	31	3.7	23	7.3	8	4.3	
Other	40	4.8	35	4.7	5	4.3	
Maternal BMI ^c^ (kg/m^2^)							
<25	476	60.6	413	60.8	63	58.9	0.217
25–29.99	170	21.6	151	22.2	19	17.8	
>30	140	17.8	115	16.9	25	23.4	
Parity							
Primiparous	388	48.2	336	48.7	52	45.2	0.489
Multiparous	417	51.8	354	51.3	63	54.8	
**Child characteristics**							
Child age (mean SD)	13.1	0.8	13.1	0.8	13.0	0.7	0.660 ^d^
Infant sex							
Boy	455	54.7	395	55.3	60	50.8	0.366
Girl	377	45.3	319	44.7	58	49.2	
Milk feeding method							
Breastmilk	219	26.3	201	28.3	18	15.3	<0.001
Breastmilk and formula	68	8.2	59	8.3	9	7.6	
Infant formula	314	37.7	275	38.5	39	33.1	
Other	231	27.8	179	25.1	52	44.1	

^a^ Chi Square *p* value; ^b^ Index of Relative Socio-Economic Advantage and Disadvantage (IRSAD) where decile 1 = most disadvantaged and decile 10 = most advantaged; ^c^ Body Mass Index; ^d^ Independent *t*-test.

**Table 2:** Nutrient intakes of children ^a^ (mean age 13.1 months) and comparison with selected Nutrient Reference Values for children aged 1–3 years.

Average Intake/Day	Mean	SD	25	75	EAR	<EAR%	UL	>UL%
Total mass (kg)	1179	393	993	1322				
Energy (kJ)	3803	714	3297	4378				
Protein (g)	36	11	28	44	12.0	0.0		
Total carbohydrate (g)	107	23	89	123				
Sugar (g)	63	17	51	73				
Total fat (g)	36	9	29	42				
Saturated fat (g)	16.5	5.1	12.8	20.1				
Polyunsaturated fat (g)	4.2	1.6	3.1	5.0				
Monounsaturated fat (g)	12.3	3.4	10.0	14.3				
Dietary fibre (g)	9.6	4.0	6.7	11.8				
Water (g)	1013	386	809	1100				
Sodium (mg)	725	324	487	916			1000	18.2
Potassium (mg)	1467	433	1171	1745				
Calcium (mg)	650	235	481	812	360	12.6	2500	0.0
Phosphorous (mg)	736	229	573	888	380	6.3	3000	0.0
Magnesium (mg)	137	38	110	164	65	2.0		
Iron (mg)	7.1	4.0	4.2	9.0	4	22.5	20	1.5
Zinc (mg)	5.4	1.5	4.4	6.4	2.5	2.2	7	14.1
Vitamin A RE ^b^ (µg)	726	492	487	851	210	0.4	NA ^c^	
Thiamin (mg)	0.8	0.5	0.5	1.0	0.4	14.4		
Riboflavin (mg)	1.4	0.6	1.0	1.8	0.4	2.1		
Niacin equivalents (mg)	16.6	5.5	12.8	19.8	5	0.0	NA ^d^	
Folate (µg)	321	130	226	404	120	3.1	NA ^d^	
Vitamin C (mg)	61	46	36	80	25	14.0		

NA—not applicable. ^a^ Children with plausible energy intakes; ^b^ Retinol equivalents; ^c^ Upper limit refers to vitamin A from retinol; ^d^ Upper limit refers to intake from supplements.

**Table 3:** Comparison of the mean nutrient intakes of children ^‡^ by milk feeding group.

	Milk Feeding Method														
	Breastmilk (*n* = 201)				Combination—Breastmilk & Formula ^§^ (*n* = 59)					Formula ^§^ (*n* = 275)				Other (*n* = 179)	
Average intake/day	Mean	SD	% CF	% BM	Mean	SD	% CF	% BM	% F	Mean	SD	% CF	% F	Mean	SD
Total mass (g)	1102 ^a^	263	67	33	1214	1047	58	21	21	1186	264	62	38	1240 ^a^	253
Energy (kJ)	3708 ^a^	747	72	28	3557 ^b^	783	62	20	18	3723 ^c^	656	66	34	4111 ^a,b,c^	646
Protein (g)	33 ^a^	10	84	16	30 ^b,c^	10	73	12	15	35 ^b,d^	9	74	26	45 ^a,c,d^	9
Total carbohydrate (g)	100 ^a,b^	25	75	25	102	21	65	16	19	110 ^a^	21	66	34	111 ^b^	22
Total fat (g)	38 ^a^	9	60	40	35	12	49	30	21	33 ^a,b^	8	58	42	38 ^b^	9
Na (mg)	700 ^a,b^	393	89	11	546 ^a,c,d^	183	79	8	13	687 ^c,e^	279	78	22	872 ^b,d,e^	284
Ca (mg)	467 ^a,b^	175	72	28	522 ^c,d^	191	50	17	33	703 ^a,c,e^	177	50	50	815 ^b,d,e^	229
Fe (mg)	5.0 ^a,b^	3.3	97	3	7.8 ^a,c,d^	4.3	68	1	31	9.3 ^b,c,e^	3.1	50	50	5.9 ^d,e^	4.2
Zinc (mg)	4.2 ^a,b,c^	1.2	84	16	5.1 ^a,d,e^	2	63	10	27	6.2 ^b,d,f^	1.2	56	44	5.6 ^c,e,f^	1.2
Thiamin (mg)	0.7 ^a,b^	0.5	89	11	0.7 ^c^	0.3	72	6	22	0.9 ^a,c^	0.4	64	36	0.9 ^b^	0.6
Vitamin C (mg)	52 ^a,b,c^	21	62	38	69 ^a,d^	30	44	20	36	83 ^b,e^	58	40	60	36 ^c,d,e^	30

^‡^ Children with plausible intakes; ^§^ Infant or toddler formula, CF = Complementary foods and beverages, BM = Breastmilk, F = Infant or toddler formula; Shared superscript letters indicate significant between group differences in the mean intake of that nutrient.

**Table 4:** Association between milk feeding method and intakes of selected ^‡^ micronutrients below the estimated average requirement (EAR) or above the upper level (UL) of intake.

	Total (*n* = 714)		Breastmilk (*n* = 201)		Formula (*n* = 334)		Other (*n* = 179)	
Calcium								
Number (%) below EAR	90	(12.6)	64	(31.8)	19	(5.7)	7	(3.9)
Adjusted ^#^ odds ratio (95% CI)			Ref		0.13	(0.07–0.22)	0.09	(0.04–0.19)
Iron								
Number (%) below EAR	161	(22.5)	98	(48.8)	11	(3.3)	52	(29.1)
Adjusted ^#^ odds ratio (95% CI)			Ref		0.04	(0.02–0.07)	0.42	(0.27–0.64)
Thiamin								
Number (%) below EAR	103	(14.4)	62	(30.8)	19	(5.7)	22	(12.3)
Adjusted ^#^ odds ratio (95% CI)			Ref		0.13	(0.07–0.23)	0.30	(0.17–0.51)
Vitamin C								
Number (%) below EAR	100	(14.0)	15	(7.5)	6	(1.8)	79	(44.1)
Adjusted ^#^ odds ratio (95% CI)			Ref		0.23	(0.09–0.59)	9.64	(5.27–17.62)
Zinc								
Number (%) above UL	101	(14.1)	4	(2.0)	75	(22.5)	22	(12.3)
Adjusted ^#^ odds ratio (95% CI)			Ref		13.5	(4.85–37.66)	6.35	(2.14–18.85)
Sodium								
Number (%) above UL	130	(18.2)	33	(16.4)	45	(13.5)	52	(29.1)
Adjusted ^#^ odds ratio (95% CI)			Ref		0.79	(0.49–1.29)	2.10	(1.28–3.44)

^‡^ Nutrients for which 10% or more of children had intakes below the EAR or above the UL; ^#^ Adjusted for maternal age, education and infant sex.

## Supplementary Materials

The following is available online at http://www.mdpi.com/2072-6643/8/8/501/s1: Table S1 Comparison of characteristics of participants and non-participants and South Australian pregnancy outcome data for 2013.

## Abbreviations

The following abbreviations are used in this manuscript:

AIFG	Australian Infant Feeding Guidelines
CI	Confidence interval
EAR	Estimated average requirement
IRSAD	Index of Relative Socio-Economic Advantage and Disadvantage
NRV	Nutrient reference value
OR	Odds ratio
RE	Retinol equivalents
UL	Upper level of intake
