# Supplementary material for: A Comparison by Milk Feeding Method of the Nutrient Intake of a Cohort of Australian Toddlers
Source: Nutrients. 2016 Aug 16;8(8):501. doi: 10.3390/nu8080501 (PMC4997414; doi:10.3390/nu8080501)
Supplement: Supplementary file 1 [file nutrients-08-00501-s001.docx]

Supplementary Materials: A Comparison by Milk Feeding Method of the Nutrient Intake of a Cohort of Australian Toddlers

Jane Scott, Kristina Davey, Ellen Ahwong, Gemma Devenish, Diep Ha and Loc Do

**Table S1.** Comparison of characteristics of participants and non-participants and South Australian pregnancy outcome data for 2013 [1].

|  | **Non-Participants  (*n* = 1091)** | | | **Participants  (*n* = 832)** | | | **SA Pregnancy Outcome 2013  (*n* = 19,925)** | | |
| --- | --- | --- | --- | --- | --- | --- | --- | --- | --- |
|  | % | 95% CI | | % | 95% CI | | % | 95% CI | |
| Maternal age (years) |  |  |  |  |  |  |  |  |  |
| <25 | 20.6 | 18.2 | 23.0 | 8.9 | 4.8 | 13.0 | 17.8 | 17.2 | 18.4 |
| 25–29 | 31.9 | 29.1 | 34.7 | 31.2 | 23.6 | 38.8 | 29.4 | 28.7 | 30.1 |
| 30+ | 47.5 | 44.5 | 50.5 | 59.8 | 49.3 | 70.3 | 52.7 | 51.7 | 53.7 |
| Maternal education |  |  |  |  |  |  |  |  |  |
| ≤year 12 | 59.4 | 56.5 | 62.3 | 43.3 | 34.3 | 52.3 | N/A |  |  |
| >year 12 | 40.6 | 37.7 | 43.5 | 56.7 | 46.4 | 67.0 |  |  |  |
| ISRAD |  |  |  |  |  |  |  |  |  |
| 1 | 70.8 | 68.1 | 73.5 | 29.2 | 21.8 | 36.6 | N/A |  |  |
| 2 | 56.9 | 53.9 | 59.9 | 43.1 | 34.1 | 52.1 |  |  |  |
| 3 | 53.0 | 50.0 | 56.0 | 47.0 | 37.6 | 56.4 |  |  |  |
| 4 | 54.8 | 51.8 | 57.8 | 45.2 | 36.0 | 54.4 |  |  |  |
| 5 | 46.5 | 43.5 | 49.5 | 53.5 | 43.5 | 63.5 |  |  |  |
| Mother’s country of birth |  |  |  |  |  |  |  |  |  |
| Australia/New Zealand | 66.6 | 63.8 | 69.4 | 74.1 | 62.4 | 85.8 | 76.5 | 75.4 | 77.6 |
| China | 2.3 | 1.4 | 3.2 | 4.4 | 1.5 | 7.3 | 2.2 | 2.0 | 2.4 |
| India | 10.4 | 8.6 | 12.2 | 6.0 | 2.7 | 9.3 | 4.0 | 3.7 | 4.3 |
| UK | 3.8 | 2.7 | 4.9 | 3.7 | 1.1 | 6.3 | 2.5 | 2.3 | 2.7 |
| Rest of Asia | 10.6 | 8.8 | 12.4 | 6.9 | 3.3 | 10.5 | 6.4 | 6.1 | 6.7 |
| Other | 6.3 | 4.9 | 7.7 | 4.8 | 1.8 | 7.8 | 5.8 | 5.5 | 6.1 |
| Parity |  |  |  |  |  |  |  |  |  |
| Primiparous | 44.7 | 41.7 | 47.7 | 48.2 | 38.6 | 57.8 | 43.3 | 42.4 | 44.2 |
| Multiparous | 55.3 | 52.3 | 58.3 | 51.8 | 41.8 | 61.8 | 56.7 | 55.7 | 57.7 |
| Maternal BMI (*n* = 16,222) |  |  |  |  |  |  |  |  |  |
| <25 | 52.8 | 49.7 | 55.9 | 60.6 | 49.7 | 71.5 | 48.1 | 47.1 | 49.1 |
| 25–29.99 | 24.9 | 22.2 | 27.6 | 21.6 | 15.1 | 28.1 | 27.9 | 27.1 | 28.7 |
| ≥30 | 22.3 | 19.7 | 24.9 | 17.8 | 11.9 | 23.7 | 24.0 | 23.3 | 24.7 |
| Infant Sex (*n* = 20,263) |  |  |  |  |  |  |  |  |  |
| Male | 51.7 | 48.7 | 54.7 | 54.6 | 44.5 | 64.7 | 52.0 | 51.1 | 52.9 |
| Female | 48.3 | 45.3 | 51.3 | 45.4 | 36.2 | 54.6 | 48.0 | 47.1 | 48.9 |

References

1. Scheil, W.; Jolly, K.; Scott, J.; Catcheside, B.; Sage, L.; Kennare, R. *Pregnancy Outcome in South Australia 2013*; Pregnancy Outcome Unit SA Health, Ed.; Government of South Australia: Adelaide, Australia, 2015.
